# Relationship between fetal biometric assessment by ultrasonography and neonatal lamb vitality, birth weight and growth

**DOI:** 10.21451/1984-3143-AR2019-0006

**Published:** 2019-11-18

**Authors:** Camila Infantosi Vannucchi, Gisele Almeida Lima Veiga, Liege Cristina Garcia Silva, Cristina Fátima Lúcio

**Affiliations:** 1 Universidade de São Paulo, Faculdade de Medicina Veterinária e Zootecnia, Departamento de Reprodução Animal, São Paulo, SP, Brasil

**Keywords:** biparietal diameter, fetal ultrasonography, neonatal vitality, ovine

## Abstract

Ultrasonographic examination of pregnant ewes can enable the identification of perinatal abnormalities and establish prenatal assistance responsible for minimizing morbidity and perinatal mortality. Therefore, we aimed to evaluate the feasibility of a fetal biometric analysis by ultrasonography to predict neonatal vitality and lamb growth during the first month of life. A longitudinal study was conducted with 13 healthy ewes, subjected to ultrasonographic examination every 15 days from 60^th^ day of pregnancy until lambing, evaluating thoracic diameter, abdominal diameter, biparietal diameter, humerus, femur and placentome length. At birth, 22 lambs were assessed through Apgar score at 5 minutes and after 1 hour. Measurement of body weight was also carried out immediately at birth and weekly during 30 days after birth. Thoracic diameter showed a significant increase between 91-105 days and 121-135 days. Conversely, abdominal diameter had a progressive growth until 106-120 days, and then, a steady development was observed. Biparietal diameter showed progressive growth only towards days 91 and 105. For the humerus length, we verified a significant increase between 106-120 days and 121-135 days, remaining unaltered onwards; while femur length continued to grow until lambing. The linear regression analysis between birth weight and biparietal diameter at 60-75 days was high (R^2^=0.96; P<0.0001; coefficient of variability of 3.3%). In conclusion, ultrasonographic analysis of fetal biparietal diameter at mid-pregnancy can be used as a predictor of lamb weight at birth. Moreover, assessment of femur length at final pregnancy can be employed for fetal and neonatal development estimation.

## Introduction

Real-time ultrasound is widely employed for pregnancy diagnosis in sheep. Among several advantageous, it is possible to identify twin pregnancies, especially in early stages, estimate gestational age and fetal growth, in addition to evaluating placental development (Kelly et al., 1987; Greenwood et al., 2002). Ultrasound can detect fetal movements and heartbeats accurately in advanced pregnancy, as well as the vitality and fetal growth (Gonzáles de Bulnes et al., 1998; Scott and Gessert, 2000). Recently, Silva et al. (2018) showed that qualitative analysis of fetal-maternal structures through ultrasonography can accurately estimate gestational age, as well as fetal development in sheep. Hence, ultrasonographic examination of the pregnant ewe can enable the identification of perinatal abnormalities and establish a prenatal assistance responsible for minimizing morbidity and perinatal mortality. However, the systematic approach towards the prenatal control in ewes and the neonatal lamb is still lacking.

The evaluation of fetal growth and development can be accomplished primarily through fetal biometry, which consists mainly of ultrasonographic measurements of fetal structures, as well as of the placental unit. Fetal biometry also allows estimating gestational age and time of lambing (Manning, 1999). In sheep, there are just a few accurate methods to estimate gestational age, especially when the mating data is unknown. Therefore, ultrasonographic fetal biometry may facilitate lambing assistance and accurate control of fetal welfare and neonatal survival rates (Greenwood et al., 2002). Ultrasonographic examination can allow for the analysis of fetal viability, growth, size, number, age and sex, including placental development (Fthenakis et al., 2012).

The main measures taken in sheep are the cerebrospinal coccygeal length, biparietal diameter, circumference and area of the skull, diameter of the thorax and abdomen area and length of long bones such as femur, humerus, tibia and metacarpus (Jones and Reed, 2017). Greenwood et al. (2002) used the measurement of fetal bones by ultrasonography as a tool to predict ewe´s gestational age. For such analysis, the measurement of the length of metacarpus and the biparietal diameter are necessary, since they are considered bones of different allometric growth and high correlation with gestational age. Additionally, the femur length is considered a sensitive and accurate variable to estimate fetal growth and development (Honarvar et al., 2001).

Vitality evaluation of the neonatal lamb at birth can be performed by taking into account the Apgar score (Vannucchi et al., 2012), variation in birth weight (Gardner et al., 2007) and neonatal behavior (Dwyer et al., 2005). The evaluation of neonatal viability by using the Apgar score is a practical method of lamb clinical analysis soon after birth. In addition, the variation in birth weight of lambs is of great importance for long-term health as restricted growth lambs are associated with increasing neonatal mortality (Alexander, 1974). A poor intrauterine environment can also reflect in low birth weight by improper fetal development (Gardner et al., 2007) or asymmetric fetal growth due to insults to the ewe (Jones and Reed, 2017). Moreover, lamb survival is highly related with birth weight and their ability to stand up and suckle for themselves (Dwyer et al., 2005). Although emergency lamb assistance can be employed at birth, the ideal management is to predict the newborn for which a special medical attention has to take place in order to reduce stillbirth and neonatal loss.

Therefore, we aimed to evaluate the feasibility of a fetal biometric analysis by ultrasonography to predict neonatal vitality and lamb growth during the first month of life.

## Methods

The present study complied with the ethical requirements for the use of animals in experiments, and was approved by the Bioethics Committee of the Faculty of Veterinary Medicine and Animal Science, University of São Paulo (protocol number 2039/2010).

A longitudinal study was conducted with the use of 13 healthy Santa Inês ewes. In order to assure the appropriate sample size, an analysis was conducted with the SAS Power and Sample Size 12 (SAS Institute Inc., Cary, NC, EUA). For an acceptable statistical power (at least 0.8), 13 pregnant ewes were sufficient to demonstrate significant differences in the data. A retrospective analysis showed a power of 0.99.

Females aged between 1 and 5 years and weighted between 40 and 60 kg. All ewes were pluriparous, except for one nulliparous female. Pregnant females were housed in stalls and kept under ideal conditions of light and temperature. The diet was based on hay, concentrate, mineral salt and water *ad libitum*. Ewes were naturally bred with the same ram. The gestational period was considered 24 hours after the first day of mating (Jainudeen and Hafez, 2004).

### Ultrasonographic examination

Throughout gestation, all ewes were subjected to abdominal trichotomy and were maintained at standing position for transabdominal ultrasonography. We used a linear 5 MHz dynamic B mode real-time transducer (Piemedical®, BC Maastricht, The Netherlands). Ultrasonographic examination was performed every 15 days from the 60^th^ day of pregnancy until lambing, always by only one experienced veterinary obstetrician. At the first scanning (60 days after breeding), ewes were diagnosed as pregnant, as well as having single or multiple pregnancies by identifying one or two fetal independent beating hearts, respectively. Out of 13 ewes, 9 females carried twins and 4 ewes had single pregnancies.

**Figure 1:**
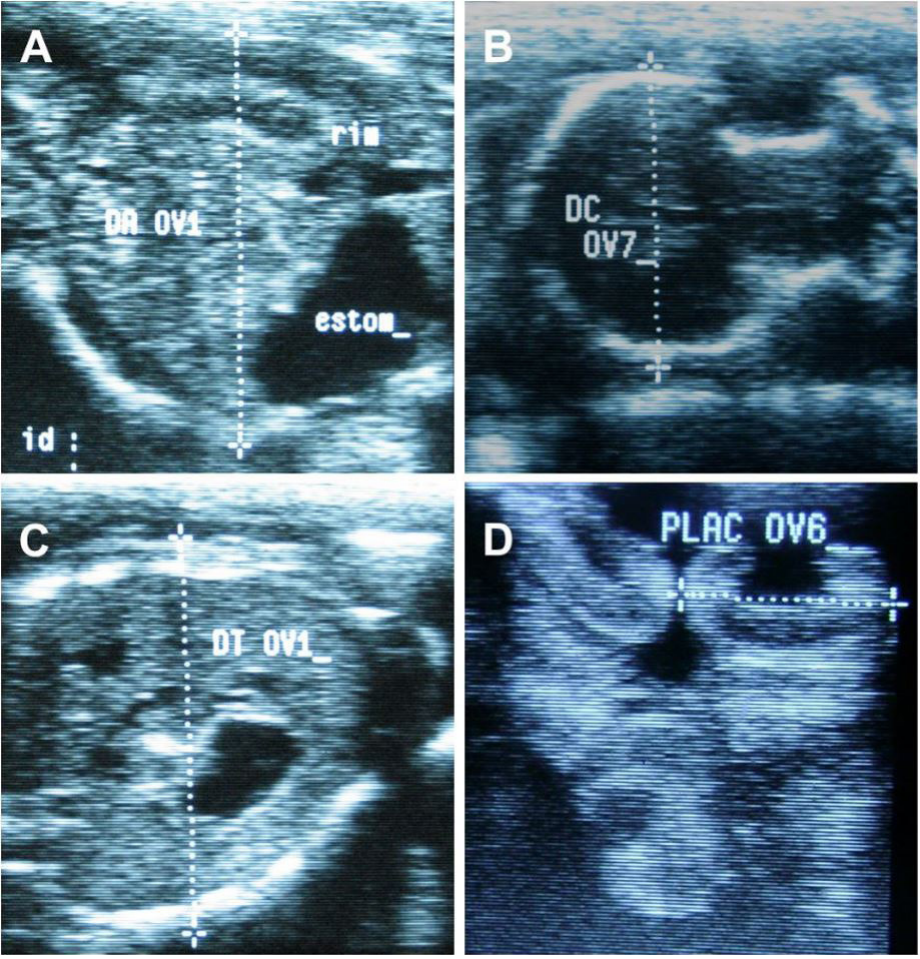
Ovine fetal biometry by B-mode ultrasonography. (A) Fetal abdomen transversely. (B) Biparietal diameter at the highest point between the parietal lobes. (C) thoracic diameter transversally at the level of the last rib. (D) placentome in longitudinal section.

Fetal biometry was evaluated by means of the thoracic diameter measurement, abdominal diameter, biparietal diameter, humerus, femur and placentome length. Fetal abdomen was analyzed transversely at the level of the umbilical cord insertion and abdominal diameter was measured (Carr et al., 2011) (Figure 1A). Biparietal diameter was measured from the highest point between the parietal lobes, perpendicular to the echogenicity produced by sagittal sutures (Greenwood et al., 2002) or the greatest distance between parietal bones behind the proximal part of the zygomatic process of the frontal bone (Barbera et al., 1995) (Figure 1B). The thoracic diameter measurement was determined transversally at the level of the last rib and the fetal stomach (Gonzáles de Bulnes et al., 1998) (Figure 1C). In order to analyze placentome length, 3 placentomes were randomly selected and measured in longitudinal section (Figure 1D). The mean length was then calculated. Femur and humerus length were measured as the length of the calcified shaft of both bones (Barbera et al., 1995).

### Neonatal lamb evaluation

At the beginning of the vaginal labor, females were continuously monitored and one fetus in posterior presentation dystocia was excluded from this experiment. Immediately at birth, newborns were dried and vigorously rubbed at chest area. Then, oral and nasal cavities were gently aspirated with a urethral probe coupled to a secretion aspirator.

At birth, 5 minutes and after 1 hour of birth, 22 lambs were assessed through the Apgar score (0-10), according to Vannucchi et al. (2012), always by the same two experienced veterinary obstetrician. The measurement of body weight was also carried out immediately at birth, before suckling, using a digital scale. During the period of 30 days after birth, lambs were submitted to weekly measurement of body weight, in a digital scale. All lambs were kept with their mothers throughout the experimental period and were allowed to freely suckle.

### Statistical analysis

All data were evaluated using the SAS System for Windows (SAS Institute Inc., Cary, NC, USA). The effect of time of evaluation was estimated by the repeated measures analysis of variance (Mixed Procedure of SAS). Differences between time were analyzed using parametric and non-parametric tests, according to the residual normality (Gaussian distribution) and variance homogeneity. Whenever one of these assumptions was not respected data were transformed. If transformations were not successful, non-parametric tests were employed. To compare the moments of observation the Least Significant Difference (LSD) test was used.

Response variables were also submitted to a Pearson Correlation Analysis. Correlation coefficients were used to measure the degree of relationship between continuous variables. Additionally, linear regression model was applied to evaluate the association between birth weight and ultrasonographic fetal biometry variables. Linear regression analyses were also used to generate equations for the estimation of lamb birth weight.

Results were described as untransformed means ± SE. Statistical differences between classificatory variables for a certain response variable were considered to occur if P < 0.05.

## Results

**Figure 2:**
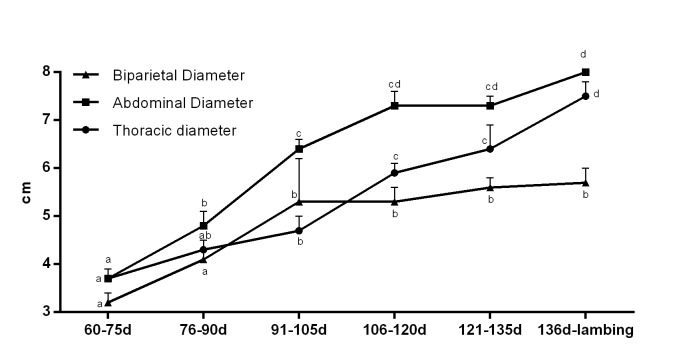
Thoracic, abdominal and biparietal diameters (cm) of ovine fetuses throughout pregnancy (n=13). ^a-d^indicate significant differences between time (P<0.05).

The thoracic diameter showed a significant increase between 91-105 days and 121-135 days, during which the thoracic area developed mostly (Table 1 and Figure 2). Conversely, abdominal diameter had a progressive growth until 106-120 days, and then, a steady development was observed (Table 1 and Figure 2). The biparietal diameter showed a progressive growth only towards days 91 and 105, from which no further increase was observed (Table 1 and Figure 2).

**Table 1:** Fetal lamb biometric measurements (cm) throughout gestation until lambing.

	60-75d	76-90d	91-105d	106-120d	121-135d	136d-lambing
Thoracic diameter	3.7 ± 0.2^a^	4.3 ± 0.2^ab^	4.7 ± 0.3^b^	5.9 ± 0.2^c^	6.4 ± 0.5^c^	7.5 ± 0.3^d^
Abdominal Diameter	3.7 ± 0.2^a^	4.8 ± 0.3^b^	6.4 ± 0.2^c^	7.3 ± 0.3^cd^	7.3 ± 0.2^cd^	8 ± 0.4^d^
Biparietal Diameter	3.2 ± 0.2^a^	4.1 ± 0.2^a^	5.3 ± 0.9^b^	5.3 ± 0.3^b^	5.6 ± 0.2^b^	5.7 ± 0.3^b^
Humerus length	2.2 ± 0.1^a^	3.1 ± 0.2^ab^	3.8 ± 0.3^b^	4.2 ± 0.3^b^	5.4 ± 0.4^c^	5.5 ± 0.6^c^
Femur length	2.6 ± 0.1^a^	3.4 ± 0.1^b^	4.3 ± 0.3^c^	4.8 ± 0.2^c^	5.9 ± 0.3^d^	6.5 ± 0.1^e^
Placentome length	3.1 ± 0.1	3.6 ± 0.2	3.4 ± 0.2	3.2 ± 0.1	3 ± 0.1	3.3 ± 0.2

^a-d^indicate significant differences between time (P<0.05).

**Figure 3:**
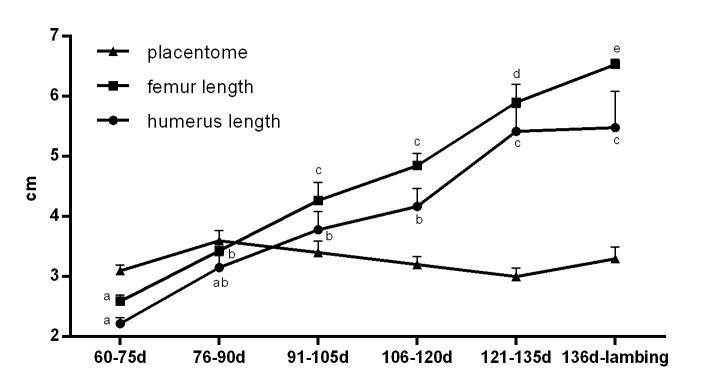
Humerus, femur and placentome lengths (cm) of ovine fetuses throughout pregnancy (n=13). ^a-e^indicate significant differences between time (P<0.05).

Placentome length changed slightly throughout pregnancy, keeping up with similar size from the 60^th^ day of pregnancy onwards (Table 1 and Figure 3). Both femur and humerus length showed a progressive growth throughout pregnancy (Table 1 and Figure 3). For the humerus length, we verified a significant increase between 106-120 days and 121-135 days, remaining unaltered onwards; while femur length continued to growth until lambing (Table 1 and Figure 3).

At birth, lamb’s Apgar score (6.3 ± 1.4) was significantly lower when compared to those of 5 min (8.8 ± 0.9) and 60 min (9.9 ± 0.3). Neonatal lambs have evolved to satisfactory Apgar score (>7) already at 5 minutes after birth. At 60 minutes, the Apgar score was statistically superior to previous moments of evaluation.

The birth weight of lambs born from twin pregnancies was 3.17 Kg ± 0.64 being significantly smaller (P<0.01) in relation to lambs of single pregnancies (4.4 Kg ± 0.8). No difference in birth weight was evidenced between male (3.3 Kg ± 0.9) and female (3.1 Kg ± 0.6) lambs. In addition, after one month of life, lambs from twin pregnancy weighted 9.24 Kg ± 1.44, significantly inferior (P<0.01) to lambs from single pregnancy (12.97 Kg ± 0.21).

There was a positive correlation between biparietal diameter at 60-75 days of pregnancy and birth weight (r=0.98; P<0.0001), as well as between abdominal diameter at 60-75 days and birth weight (r=0.67; p=0.06). The femur length at days 136 until lambing correlated positively (r=0.77; p=0.07) with birth weight. The regression analysis between birth weight and biparietal diameter at 60-75 days (R^2^=0.96; P<0.0001; coefficient of variability of 3.3%) gendered the following linear regression equation: *Birth weight = -1.8933 + 1.7249 x biparietal diameter 60-75 days.*

No correlation was observed between the Apgar vitality score and lambs birth weight.

## Discussion

In the present manuscript we aimed to analyze ovine fetal biometric parameters by ultrasonography in order to predict lamb birth weight and growth within the first month of life. Although evaluation of fetal organs and bone structures are currently used to estimate gestational age (Jones and Reed, 2017), no previous report is available to predict newborn weight and development during the neonatal period. All real-time ultrasonographic measurements were feasible from 60 days of pregnancy onwards, although twin pregnancies hindered an accurate identification of individual structures of fetal biometry, notably femur and humerus length. On the other hand, according to Schrick and Inskeep (1993) and Gonzáles de Bulnes et al. (1998), fetal growth pattern is irrespective of multiple pregnancies, since the development within fetuses is uniform and well-correlated with gestational age (Haibel, 1988).

Placentome size remained constant from initial pregnancy (60 days) towards lambing, suggesting an initial placental maturation that maintained unchanged throughout pregnancy. Conversely, Lekatz et al. (2013) showed progressive placentome diameter increase from day 40 to 80 of gestation, and then remained unchanged or even diminished at day 108. Although placentome length has little relation with pregnancy progression (Doizé et al., 1997), Kelly et al. (1987) and Gonzáles de Bulnes et al. (1998) stated that placentome size varies greatly according to the degree of placental development, being an interesting measurement for the evaluation of placental integrity in sheep.

Regarding the biparietal diameter, we observed a significant increase until mid-pregnancy (91-105 days), from which it remained unchanged. In fact, biparietal diameter reaches 49% of its total growth at mid-pregnancy in sheep, showing a deceleration of development from the 75^th^ to the 133^th^ day of pregnancy (Barbera et al., 1995). Cephalic diameter and length are considered good predictors of fetal development, with high correlation with gestational age, mainly during the second trimester of pregnancy (Haibel and Perkins, 1989; Santiago-Moreno et al., 2005). Interestingly, we found a high correlation between biparietal diameter at mid-pregnancy (60-75 days) and birth weight, being an excellent predictor of the expected lamb size at birth. Conversely, biparietal diameter at advanced gestational ages had lower accuracy to estimate fetal development and lamb birth weight, albeit a less pronounced variation from 106^th^ onwards. In addition, biparietal diameter in the third trimester is limited by the cranial position of the fetus towards the xiphoidal region of the dam (Jones and Reed, 2017). Ideally, the identification of the fetuses is recommended to be done about 75 days from first introduction of the ram to the flock (Buckrell, 1988). Therefore, scanning ewes at early stages of pregnancy is more accurately performed for the purpose of both pregnancy diagnosis and fetal biometry for the prediction of lamb birth weight.

Our study shows a progressive increase in abdominal diameter throughout pregnancy, more pronounced until 91-105 days. Similarly, Barbera et al. (1995) stated that the abdominal circumference develops in 36% of its estimated length at birth until middle of pregnancy in sheep and showed linear growth from 50 to 130 days. In fact, Rurak and Wittman (2013) showed diminished rate of increase in fetal abdominal diameter with advancing gestation, which can be attributed to progressive decrease in fetal oxygen delivery. However, we observed a moderate correlation (r=0.67; p=0.06) between abdominal diameter at 60-75 days of pregnancy and lamb birth weight. On the other hand, abdominal diameter at late pregnancy strongly correlates with physical measurements of lambs at birth, in special with liver weight, being a reflection of fetal nutritional status (Carr et al., 2011). Therefore, the use of abdominal diameter to predict birth weight of lambs can be inaccurate in different nutritional management conditions of pregnant ewes. Hence, we suggest that the measurement of abdominal diameter can be used alternatively to the biparietal diameter to predict the weight of neonatal lambs at birth.

The thoracic diameter showed a pronounced increase after the 105^th^ day of pregnancy. However, we did not find a correlation with birth weight, thus, not being a feasible predictor of fetal development at term pregnancy, although thoracic measurement is reported to be highly correlated with fetal age and weight (Gonzáles de Bulnes et al., 1998; Sergeev et al., 1990). In order to estimate gestational age in mouflons, it is more accurate to scan ewes for thoracic diameter at the interval of gestation of 25–109 days (Santiago-Moreno et al. 2005). On the other hand, fetal structures are not easily identified at advanced stages of pregnancy, making it difficult to obtain an accurate measurement of the thoracic diameter. In fact, the accuracy of scanning ewes at a very late stage (101–133 days) of gestation is low for determining the exact number of fetuses (Fridlund et al., 2013).

Femur and humerus lengths presented a progressive and significant increase throughout pregnancy, similarly to previous findings of Barbera et al. (1995), for whom femur and tibia rate of growth are the slowest among biometric parameters. In addition, we showed that the length of the femur at 136 days of pregnancy until lambing has a high correlation (r=0.77; p=0.07) with the birth weight of lambs. However, Gonzáles de Bulnes et al. (1998) stated that the difficulty in scanning the entire femur transversally or longitudinally accounts for a low correlation with gestational age. In addition, the growth of fetal long bones is negatively affected by maternal malnutrition during pregnancy (Osgerby et al., 2002). Thus, in spite of the high correlation with fetal development, measurement of femur length can be affected by technical impairment and ewe’s management. On the other hand, only the femur length and thoracic diameter presented a significant increase from 135^th^ day of pregnancy towards lambing, period in which it is possible to observe a fast fetal growth (Osgerby et al., 2002). Therefore, although none of the biometric parameters at final pregnancy correlated with lamb’s weight at birth, analyzing the growth rate of the femur and thoracic diameter can drive information on the expected increase in fetal development at final stages of pregnancy and can suggest the occurrence of intrauterine growth restriction.

The birth weight of lambs of twin pregnancies was significantly inferior to lambs of single pregnancies, which is also reflected on postnatal development and growth, since twin lambs weighted less than non-twin lambs after 1 month. Fetal growth of twin or triplet lambs is proved to be physiologically restricted with advancing gestation, leading to low birth weight of lambs with multiple fetuses pregnancies (Rurak and Wittman, 2013). In fact, Sergeev et al. (1990) showed that the rate of increase in thoracic depth of twins decreased with advancing gestation. However, irrespective of birth weight, the Apgar score indicated low initial neonatal vitality, despite showing appropriate recovery after 5 minutes. Hence, neonatal lambs exhibit satisfactory ability to adapt to the external environment, despite the initial depression and size of birth.

In conclusion, the ultrasonographic analysis of fetal biparietal diameter at mid-pregnancy (60-75 days) is an accurate predictor of lamb weight at birth and the assessment of femur length at final pregnancy can be employed as guide for the diagnosis of fetal restriction growth. Both analysis allow for the evaluation of fetal development and the estimative of neonatal development, thus predicting the need of a differential assistance towards lambing and to underdeveloped neonatal lambs. However, further studies on uterine and fetal vascularization, such as uterine artery and fetus cerebral artery hemodynamics may provide a better understanding on fetal development and well-being.
